# Vaborbactam increases meropenem susceptibility in *Pseudomonas aeruginosa* clinical isolates displaying MexXY and AmpC upregulation

**DOI:** 10.1128/msphere.00162-23

**Published:** 2023-09-28

**Authors:** Mariana Castanheira, Timothy B. Doyle, Cory M. Hubler, Sean DeVries, Dee Shortridge

**Affiliations:** 1 JMI Laboratories, North Liberty, Iowa, USA; Antimicrobial Development Specialists, LLC, Nyack, New York, USA

**Keywords:** *P. aeruginosa*, β-lactam/β-lactamase inhibitor combinations, efflux

## Abstract

To evaluate the resistance mechanisms among *Pseudomonas aeruginosa* clinical isolates exhibiting meropenem (MEM) MIC values higher than meropenem-vaborbactam (MEV). *P. aeruginosa* clinical isolates collected in US hospitals from 2014 to 2019 were susceptibility tested. Whole-genome and transcriptome sequencing were performed. Results were analyzed for strain typing, acquired β-lactamases, and mutations in chromosomal genes; gene expression was measured for known β-lactam resistance contributors. Results were compared to a control group of 10 *P*. *aeruginosa* isolates displaying MIC values at 8 mg/L for meropenem ± vaborbactam (MEM = MEV). Out of 88 isolates displaying MEM > MEV, 33 (37.5%) isolates had reproducibly lower MIC values for meropenem-vaborbactam compared to meropenem when retested. The expression of *mexX*, *mexY*, *mexZ*, and *ampC* was significantly greater among a higher percentage of the MEM > MEV isolates. Furthermore, the association of *mexXY* and *ampC* overexpression was detected in 17/33 MEM > MEV isolates and only 1/10 MEM = MEV isolate. In addition, the *Pseudomonas*-derived cephalosporinase amino acid substitution R79Q was detected among 33.3% of the isolates displaying MEM > MEV, and none of the isolates displayed MEM = MEV. Other resistance mechanisms were not observed or were equally observed in both groups. In rare cases, vaborbactam plays a role in lowering the meropenem MIC values in *P. aeruginosa* clinical isolates likely due to the inhibition of the AmpC gene that was overexpressed in the presence of upregulation of MexXY with or without alterations in the AmpC gene.

**IMPORTANCE:**

*Pseudomonas aeruginosa* isolates are intrinsically resistant to multiple antimicrobial agents and meropenem is an important therapeutic option to treat infections caused by this organism. Meropenem-vaborbactam activity is similar to that of meropenem alone against *P. aeruginosa* isolates. Isolates belonging to this species that display lower meropenem-vaborbactam compared to meropenem are rare. We initiated this study to understand the resistance mechanisms that could lead to lower meropenem-vaborbactam MIC values when compared to meropenem alone. We documented that isolates displaying lower meropenem-vaborbactam exhibited overexpression of MexXY and AmpC. In addition, isolates displaying the R79Q PDC (AmpC) mutation were more likely to display lower meropenem-vaborbactam when compared to isolates displaying the same MIC values for these agents.

## INTRODUCTION

Meropenem is an important option for the treatment of infections caused by *Pseudomonas aeruginosa* due to its safety profile and stability against common resistance mechanisms (1, 2), including most acquired β-lactamases, but not carbapenemases (3). Meropenem has greater stability against the constitutive cephalosporinase (AmpC) from *P. aeruginosa* when compared to imipenem and other antipseudomonal agents (4). Moreover, meropenem, unlike imipenem, is not as affected by OprD deficiency. Despite its stability against described mechanisms, meropenem can be extruded from the cell by the RND-family efflux systems MexAB-OprM and MexXY (5). These mechanisms alone might not lead to meropenem resistant MIC values, but in combination with other intrinsic and acquired resistance mechanisms, these mechanisms confer meropenem MIC values in the resistant categories.

The interplay of resistance mechanisms has been pointed out as the main cause of carbapenem resistance among *P. aeruginosa* clinical isolates. In a study evaluating 33 carbapenem-resistant *P. aeruginosa* isolates from eight hospitals in New York, the isolates had combinations of decreased OprD, increased AmpC expression (39.4%), or OprD decrease and overexpression of efflux (60.6%) (6). In an evaluation of carbapenem-nonsusceptible *P. aeruginosa* isolates from 14 European countries, the overexpression of AmpC, MexAB-OprM, and MexXY was noted among 37.2%, 20.1%, and 35.7% of the isolates, respectively, while OprD loss or decrease was noted among 94.9% of the isolates (7) indicating that most isolates had multiple resistance mechanisms. In a Spanish multicenter study, AmpC overexpression was prevalent among meropenem-resistant isolates; however, around 30% of the isolates also exhibited overexpression of MexAB-OprM or MexXY (8).

Meropenem-vaborbactam was approved in 2017 by the US FDA for the treatment of complicated urinary tract infections, including pyelonephritis caused by Enterobacterales isolates in adult patients. This β-lactam/β-lactamase inhibitor combination was approved by the European Medicines Agency in 2018 for the same indication, with an additional approval for *P. aeruginosa*. Meropenem-vaborbactam was approved with a prolonged infusion dose and granted a *P. aeruginosa* breakpoint of ≤8 mg/L for susceptibility by the EUCAST (9).

The activity of meropenem-vaborbactam against *P. aeruginosa* is described as the same as meropenem alone (10) as vaborbactam does not offer protection for decreased permeability or increased extrusion of meropenem from the cell. However, during the post-approval surveillance for meropenem-vaborbactam, 88/17,180 (0.5%) *P*. *aeruginosa* clinical isolates collected in US hospitals from 2014 to 2019 displayed meropenem-vaborbactam MIC values lower than the results for meropenem. In this study, we evaluated the resistance mechanisms of these isolates using 10 isolates with the same MIC values for meropenem and meropenem-vaborbactam as control isolates.

## MATERIALS AND METHODS

### Bacterial isolates and susceptibility testing

Among 17,180 *P*. *aeruginosa* collected worldwide from 2014 to 2019 as part of the meropenem-vaborbactam surveillance studies (11), 88 isolates displayed MIC values for meropenem-vaborbactam lower than meropenem (MEM > MEV) and meropenem-vaborbactam ≤8 mg/L when initially tested. These 88 isolates were submitted to confirmatory susceptibility testing against meropenem ± vaborbactam alongside 20 isolates exhibiting MIC values at 8 mg/L for meropenem and meropenem-vaborbactam (MEM = MEV) susceptibility tested as potential controls. The control isolates were selected to represent a diverse set of hospitals that participated in the surveillance study and displayed different susceptibility profiles. Species identification was confirmed when needed by matrix-assisted laser desorption ionization-time of flight mass spectrometry using the Bruker Daltonics MALDI Biotyper (Billerica, Massachusetts, USA) following the manufacturer’s instructions.

### Antimicrobial susceptibility testing

Susceptibility results were initially determined, and results were confirmed using the reference broth microdilution methods conducted according to Clinical and Laboratory Standards Institute (CLSI) procedures (12). Quality control (QC) testing was performed to ensure proper test conditions. QC strains included *Escherichia coli* ATCC 25922 and NCTC 13353, *Klebsiella pneumoniae* ATCC 700603 and ATCC BAA-1705, and *P. aeruginosa* ATCC 27853. CLSI guidelines were used for the interpretation of susceptibility results (13). Vaborbactam was provided by Melinta Therapeutics. Other agents were acquired from Sigma-Aldrich (Saint Louis, Missouri, USA) or US Pharmacopeia (Rockville, Maryland, USA).

### Characterization of β-lactam resistance mechanisms

All 33 *P*. *aeruginosa* isolates confirmed to have MEM > MEV and 10 isolates with MEM = MEV selected as control were subjected to whole-genome sequencing (WGS). Genomic libraries were constructed using the Nextera XT protocol and index kit (Illumina, San Diego, California, USA) following the manufacturer’s instructions and sequenced on a MiSeq Sequencer (Illumina) or constructed using the Illumina DNA Prep protocol with IDT for Illumina Unique Dual indexes (Illumina) and sequenced on a NextSeq 1000 Sequencer (Illumina). FASTQ format files for each sample set were assembled independently using *de novo* assembler SPAdes 3.9.0 (14) with *K*-values of 21, 33, 55, 77, and 99 and “careful mode” to reduce the number of mismatches. This process produced a FASTA format file of contiguous sequences with the best N50 value. An in-house-designed software using the target assembled sequences (15) as queries to align against numerous resistance determinants from the NCBI Bacterial Antimicrobial Resistance Reference Gene Database (https://www.ncbi.nlm.nih.gov/bioproject/PRJNA313047) was used to search for β-lactamase genes. Potential matches were generated with the criteria of >94% identity and 40% minimum coverage length (16).

Total RNA was extracted and purified from log phase bacterial cultures that displayed a cell density of optical density (OD)_600_ of 0.3 to 0.5 using the RNeasy Mini Kit in the Qiacube workstation (Qiagen, Hilden, Germany) according to the manufacturer’s instructions. Residual DNA was eliminated by treatment with RNAse-free DNase (Promega, Madison, Wisconsin, USA). Quantification of total RNA and sample quality was assessed using the RNA 6000 Pico kit on the Agilent 2100 Bioanalyzer (Agilent Technologies, Santa Clara, California, USA) according to the manufacturer’s instructions. Only preparations with acceptable RNA integrity numbers (RIN) ≥7 and/or that showed no visual degradation were used for experiments.

A total of up to 1 µg of RNA was subjected to rRNA depletion using Ribo-Zero Plus as part of the Illumina Stranded Total RNA Prep Kit with Ribo-Zero Plus (Illumina) according to the manufacturer’s instructions. The resultant rRNA-depleted RNA was then purified using RNA-specific magnetic beads and eluted in 8.5 µL of elution buffer for downstream library preparation. Whole-transcriptome RNA sequencing cDNA library preparation was performed using the Illumina Stranded Total RNA Prep, Ligation (Illumina) with eluted Ribo-Zero-treated RNA samples as input material. Library preparation was performed according to the manufacturer’s instructions, beginning with mRNA fragmentation. mRNA fragmentation was accomplished using the entire eluted Ribo-Zero-treated RNA sample (8.5 µL) combined with 8.5 µL of EPH3 (Elute, Prime, Fragment 3HC Mix). Sequencing was completed on a NextSeq 1000 Sequencer using NextSeq 1000 P2 Reagents. An independently prepared replicate of the control reference isolate (*P. aeruginosa* PAO1) was included with each sequencing run to serve as an internal control.

Differential gene expression was estimated using an RNA-seq pipeline. First, paired-end reads were trimmed, corrected, and filtered using FASTA. Quality-controlled reads were aligned to a *P. aeruginosa* PAO1 reference assembly (ASM676v1), filtered based on alignment scores, and assigned to loci to calculate per-gene counts using EDGE-pro (17). Counts were normalized across samples using the trimmed mean of *M*-values normalization, and fold change expression was calculated according to an exact test based on the quantile-adjusted conditional maximum likelihood method using edgeR (18). Synonyms and gene ontology (GO) terms were collected from UniProt to aid in interpretation.

Differences in expression were considered significant if they were ±five-fold of the expression of *P. aeruginosa* PAO1.

## RESULTS

**Fig 1:**
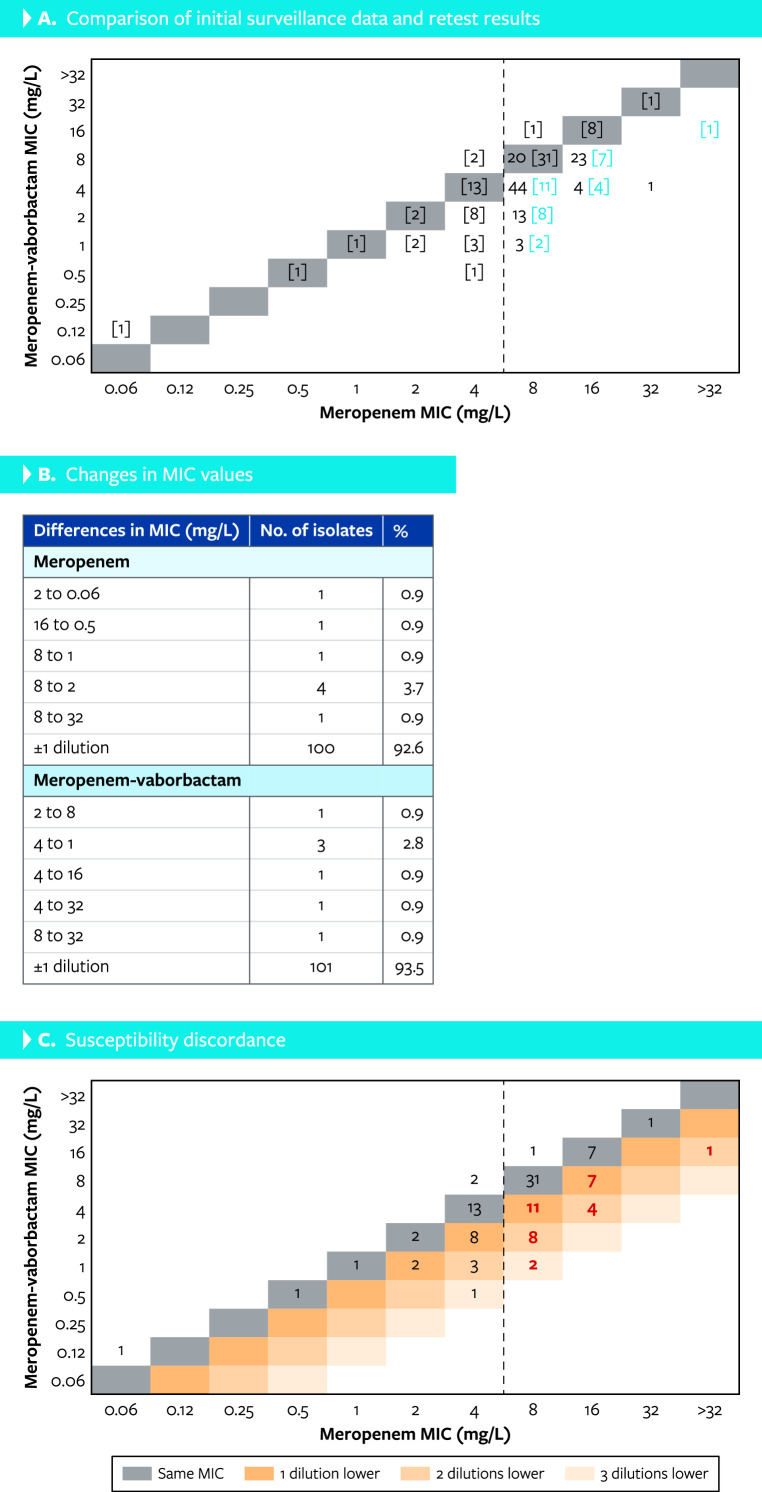
Susceptibility retesting results for 88 *P*. *aeruginosa* isolates initially displaying meropenem-vaborbactam MIC values lower than meropenem and meropenem-vaborbactam ≤8 mg/L. (**A**) Comparison of initial surveillance data and retest results in brackets for isolates displaying meropenem-vaborbactam MIC values lower than meropenem. The 33 isolates with confirmed lower meropenem-vaborbactam and exhibiting meropenem MIC values ≥8 mg/L were further tested in this study (in blue) and 10 isolates displaying an MIC of 8 mg/L for both agents. (**B**) Changes in MIC values from initial testing and retesting. (**C**) Susceptibility discordance for meropenem and meropenem-vaborbactam: 33 isolates that were further characterized are in red.

Of the 88 (0.5% of the overall isolates) *P. aeruginosa* isolates initially displaying meropenem MIC values greater than meropenem-vaborbactam and meropenem-vaborbactam ≤8 mg/L, 33 isolates were confirmed to have greater meropenem MIC results upon retesting (phenotype MEM > MEV; Fig. 1A through C). A toltal of 19 isolates displayed meropenem-vaborbactam MIC values one dilution lower than meropenem and MIC values >8 mg/L for both agents (Fig. 1C). In total, 12 and 2 isolates exhibited meropenem-vaborbactam MIC values lower than meropenem by two or three dilutions, respectively (Fig. 1C).

Among the 20 isolates that displayed MIC values at 8 mg/L for meropenem and meropenem-vaborbactam and were initially used as controls for the susceptibility testing, all 20 displayed reproducible MIC values. This MIC was selected because this is the concentration achieved with a 3 h of infusion often used for meropenem and recommended for meropenem-vaborbactam and isolates at this MIC would be considered susceptible to both agents. Ten of these isolates were randomly selected for further evaluation (phenotype MEM = MEV).

MLST results revealed a diversity of sequence types (STs) among the 43 *P*. *aeruginosa* evaluated by WGS (33 MEM > MEV and 10 MEM = MEV), and there were no predominant types in each group (Table 1). In addition, high-risk clones described among *P. aeruginosa* isolates, such as ST111 or ST235, were only detected in two isolates, one from each group. Only three isolates harbored acquired β-lactamase-encoding genes (Table 1). One MEM = MEV carried genes encoding OXA-9 and CARB-2 (also known as PSE-1). One MEM > MEV harbored *bla*_KPC-2_ and another carried *bla*_OXA-2_.

**TABLE 1:** MLST results and β-lactamases detected among *P. aeruginosa* isolates displaying the same MIC values for meropenem and meropenem-vaborbactam (MEM = MEV) compared to meropenem-resistant and meropenem-vaborbactam-susceptible isolates (MEM > MEV)

Collection number	Group	Sequence type	Acquired β-lactamases	OXA-50 family	*Pseudomonas*-derived cephalosporinase amino acid (PDC)	Amino acid changes against PDC-1	PDC Ω-loop alterations	PA5542
873972	MEM = MEV	553		OXA-486	PDC-3	T105A		I106V, E123A, S224A
875455	MEM = MEV	162		OXA-904-like	PDC-31	T105A, V205L	y	I106V, S224A, D302N
882600	MEM = MEV	298		OXA-848	PDC-16	G27D, T105A, V205L, G391A	y	V77A, I106V, S224A
885328	MEM = MEV	111	CARB-2/PSE-1, OXA-9	OXA-395	PDC-3	T105A		V17I, I106V, S224A
921883	MEM = MEV	139		OXA-488-like	PDC-39	T105A, V205L, G391A	y	T68S, I106V
933365	MEM = MEV	217		OXA-494	PDC-3	T105A		I106V, P161L
1057409	MEM = MEV	395		OXA-905	PDC-8	T105A, L176R		I106V
1097117	MEM = MEV	1667		OXA-50	PDC-3	T105A		I106V, S325T
1111577	MEM = MEV	308		OXA-488	PDC-19a-like	G27D, T105A, V205L, T316 deletion, P317 deletion, V356I, G391A	y	V77A, I106V, V112I, S224A
1120249	MEM = MEV	959		OXA-50	PDC-103	P82L, T105A		I106V, S325T
826167	MEM > MEV	2615		OXA-50	PDC-97	T105A, V239A	y	I106V, S325T
831117	MEM > MEV	245		OXA-494	PDC-5	R79Q, T105A		I106V, P161L
873504	MEM > MEV	348		OXA-494	PDC-5	R79Q, T105A		I106V, D302N
883214	MEM > MEV	236		OXA-486	PDC-31	T105A, V205L	y	I106V
883279	MEM > MEV	235	KPC-2	OXA-488	PDC-35	G27D, A97V, T105A, V205L, G391A	y	I106V, S224T
888817	MEM > MEV	2615		OXA-50	PDC-119	T105A, G242S	y	I106V, S325T
919108	MEM > MEV	260		OXA-904	PDC-117	T105A, D272N		I106V
920788	MEM > MEV	2686		OXA-494	PDC-71	R79Q, T105A, R235H	y	I106V, K111N, D302N, A363V, A412T
933620	MEM > MEV	450-like		OXA-486	PDC-5	R79Q, T105A		I106V, S325T
945162	MEM > MEV	256-like		OXA-851	PDC-31	T105A,V205L	y	V77A, I106V, S224T
945300	MEM > MEV	155		OXA-396	PDC-5	R79Q, T105A		I106V, S325T
945827	MEM > MEV	809		OXA-494	PDC-147	T105A, E198K, V205L	y	T68A, I106V
948188	MEM > MEV	480		OXA-50-like	PDC-8	T105A, L176R		I106V
1018205	MEM > MEV	17		OXA-396	PDC-5	R79Q, T105A		I106V, S224A, D302N
1018193	MEM > MEV	155		OXA-50	PDC-8	T105A, L176R		I106V, S325T
1047044	MEM > MEV	2587		OXA-395-like	PDC-1			I106V, G336S
1063866	MEM > MEV	17		OXA-50	PDC-205	T105A, L176R, H215Y, N347S	y	I106V, S224A, D302N
1063946	MEM > MEV	274		OXA-486	PDC-24	G27D, T105A, V205L, V239A, T21A, T105A, G391A		I106V
1063970	MEM > MEV	1642-like		OXA-50	PDC-6	R79Q		I106V, E123A, S224A
1063927	MEM > MEV	298	OXA-2	OXA-848	PDC-143	G24D, T105A, V205L, V239A, G391A	y	V77A, I106V, S224A
1069529	MEM > MEV	155		OXA-396	PDC-5	R79Q, T105A		I106V, S325T
1070223	MEM > MEV	3756		OXA-50	PDC-31	T105A, V205L	y	I106V, S224A
1073143	MEM > MEV	2952		OXA-50-like	PDC-31	T105A, V205L	y	I106V
1091354	MEM > MEV	258		OXA-494	PDC-8	T105A, L176R		I106V, S325T
1094266	MEM > MEV	471		OXA-396	PDC-5	R79Q, T105A		I106V, S325T
1111663	MEM > MEV	155		OXA-396	PDC-5	R79Q, T105A		I106V, S325T
1113238	MEM > MEV	1693		OXA-904	PDC-5-like	R79Q, T105A, D245G, N347S	y	I106V, S325T
1114687	MEM > MEV	2587		OXA-395-like	PDC-1			I106V, G336S
1120958	MEM > MEV	2587		OXA-395-like	PDC-1			I106V, G336S
1125996	MEM > MEV	274-like		OXA-486	PDC-24	T21A, T105A, G391A		I106V
1126967	MEM > MEV	550-like		OXA-396	PDC-3	T105A		I106V, S325T
1169244	MEM > MEV	3466		OXA-396	PDC-8	T105A, L176R		I106V, S325T
1173541	MEM > MEV	968-like		OXA-851	PDC-31	T105A, V205L	y	V77A, I106V, S224T

The variant of chromosomal AmpC from *P. aeruginosa*, *Pseudomonas*-derived cephalosporinase amino acid (PDC), was differently represented in the MEM = MEV phenotype group compared to the MEM > MEV phenotype isolates (Tables 1 and 2). PDC-5 and PDC-1 were only noted among MEM > MEV isolates. PDC-3 was more common among MEM = MEV isolates compared to the MEM > MEV isolates (four isolates and one isolate, respectively). Conversely, PDC-8 and PDC-31 were detected in a greater number of MEM > MEV phenotype isolates (four and five isolates, respectively) when compared to MEM = MEV isolates (one of each). When analyzing discrete amino acid substitutions in the PDC sequences and comparing them to PDC-1, the substitution R79Q was noted in 11 (33.3%) isolates of the MEM > MEV phenotype and was not found among the MEM = MEV isolates (Table 2). This alteration is located near the helix H-2 and was observed among the alleles PDC-5, PDC-5-like, PDC-6, and PDC-71 (19). Alterations in the PDC Ω-loop that have been associated with resistance against novel β-lactam/β-lactamase inhibitor combinations were noted among 13 (39.4%) and 4 (40.0%) isolates from the MEM > MEV and MEM = MEV groups, respectively.

**TABLE 2:** Genetic characterization results for *P. aeruginosa* isolates displaying the same MIC values for meropenem and meropenem-vaborbactam (MEM = MEV) compared to meropenem-resistant and meropenem-vaborbactam-susceptible isolates (MEM > MEV)

	Isolate count (%)	
Molecular results	MEM = MEV (*n* = 10)	MEM > MEV (*n* = 33)
Overexpression/reduced expression for *oprD*		
*mexA*	5 (50.0%)	22 (66.7%)
*mexB*	5 (50.0%)	16 (48.5%)
*oprM*	5 (50.0%)	10 (30.3%)
*mexAB-oprM*	5 (50.0%)	16 (48.5%)
*mexR*	1 (10.0%)	3 (9.1%)
*mexC*	2 (20.0%)	3 (9.1%)
*mexD*	2 (20.0%)	1 (3.0%)
*mexE*	0 (0.0%)	0 (0.0%)
*mexF*	0 (0.0%)	0 (0.0%)
*mexY*	6 (60.0%)	30 (90.9%)
*mexX*	7 (70.0%)	30 (90.9%)
*mexXY*	6 (60.0%)	30 (90.9%)
*mexZ*	5 (50.0%)	29 (87.9%)
*pdc*	3 (30.0%)	20 (60.6%)
*oprD*	1 (10.0%)	5 (15.2%)
*oxa-50*	0 (0.0%)	3 (9.1%)
*pib-1*	0 (0.0%)	2 (6.1%)
*mexXY* plus *pdc*	1 (10.0%)	17 (51.5%)
Gene sequence/sequencing alterations		
OXA-50-like		
OXA-395	1 (10.0%)	0 (0.0%)
OXA-395-like	0 (0.0%)	3 (9.1%)
OXA-396	0 (0.0%)	7 (21.2%)
OXA-486	1 (10.0%)	4 (12.1%)
OXA-488	1 (10.0%)	1 (3.0%)
OXA-488-like	1 (10.0%)	0 (0.0%)
OXA-494	1 (10.0%)	5 (15.2%)
OXA-50	2 (20.0%)	6 (18.2%)
OXA-50-like	0 (0.0%)	2 (6.1%)
OXA-848	1 (10.0%)	1 (3.0%)
OXA-851	0 (0.0%)	2 (6.1%)
OXA-904	0 (0.0%)	2 (6.1%)
OXA-904-like	1 (10.0%)	0 (0.0%)
OXA-905	1 (10.0%)	0 (0.0%)
PDC-like		
PDC-1	0 (0.0%)	3 (9.1%)
PDC-3	4 (40.0%)	1 (3.0%)
PDC-5	0 (0.0%)	8 (24.2%)
PDC-5-like	0 (0.0%)	1 (3.0%)
PDC-6	0 (0.0%)	1 (3.0%)
PDC-8	1 (10.0%)	4 (12.1%)
PDC-16	1 (10.0%)	0 (0.0%)
PDC-19a-like	1 (10.0%)	0 (0.0%)
PDC-24	0 (0.0%)	2 (6.1%)
PDC-31	1 (10.0%)	5 (15.2%)
PDC-35	0 (0.0%)	1 (3.0%)
PDC-39	1 (10.0%)	0 (0.0%)
PDC-71	0 (0.0%)	1 (3.0%)
PDC-97	0 (0.0%)	1 (3.0%)
PDC-103	1 (10.0%)	0 (0.0%)
PDC-117	0 (0.0%)	1 (3.0%)
PDC-119	0 (0.0%)	1 (3.0%)
PDC-143	0 (0.0%)	1 (3.0%)
PDC-147	0 (0.0%)	1 (3.0%)
PDC-205	0 (0.0%)	1 (3.0%)
Annotations against PDC-1		
T105A	10 (100.0%)	29 (87.9%)
V205L	4 (40.0%)	8 (24.2%)
G27D	2 (20.0%)	2 (6.1%)
G391A	3 (30.0%)	4 (12.1%)
L176R	1 (10.0%)	5 (15.2%)
R79Q	0 (0.0%)	11 (33.3%)
PDC Ω-loop alterations		
Yes	4 (40.0%)	13 (39.4%)
AmpR annotations		
G283E	5 (50.0%)	24 (72.7%)
E287G	2 (20.0%)	2 (6.1%)
M288Q	2 (20.0%)	2 (6.1%)
A290V	2 (20.0%)	2 (6.1%)
V291L	2 (20.0%)	2 (6.1%)
A293S	2 (20.0%)	2 (6.1%)
M288R	2 (20.0%)	3 (9.1%)
R244W	0 (0.0%)	3 (9.1%)
OprD disruption	9 (90.0%)	29 (87.9%)
ArmZ annotations		
L88P	10 (100.0%)	33 (100.0%)
D161G	7 (70.0%)	29 (87.9%)
H182Q	8 (80.0%)	30 (90.9%)
V243A	10 (100.0%)	33 (100.0%)
C40R	2 (20.0%)	4 (12.1%)
S112N	2 (20.0%)	1 (3.0%)
D119E	2 (20.0%)	4 (12.1%)
I237V	3 (30.0%)	2 (6.1%)
N238S	1 (10.0%)	2 (6.1%)
A352V	1 (10.0%)	2 (6.1%)
MexZ annotations		
X211S	0 (0.0%)	3 (9.1%)
X211R	0 (0.0%)	3 (9.1%)
Q95X	0 (0.0%)	3 (9.1%)
FtsI annotations		
R504C	1 (10.0%)	3 (9.1%)
G63D	0 (0.0%)	4 (12.1%)
N242S	0 (0.0%)	3 (9.1%)
P215L	0 (0.0%)	3 (9.1%)
DacB annotations		
A394P	2 (20.0%)	5 (15.2%)
A474T	0 (0.0%)	4 (12.1%)
DacC annotations		
P62L	0 (0.0%)	3 (9.1%)
NalD annotations		
T188A	1 (10.0%)	3 (9.1%)
MexR annotations		
V126E	4 (40.0%)	2 (6.1%)

Among the other intrinsic β-lactamases in *P. aeruginosa*, variations within the sequences of the OXA-50 family member and PIB-1 (PA5542) seemed to be randomly distributed among the two groups (Table 1).

An analysis of the expression levels of intrinsic genes encoding components or promoter region sequences of efflux systems, chromosomal β-lactamases, and the outer membrane protein OprD revealed alterations. However, the only differences noted among the two groups were the expression of the genes encoding PDC, MexX, MexY, and the *mexXY* regulator, MexZ. These genes were overexpressed in 20 (60.6%), 30 (90.9%), 30 (90.9%), and 29 (87.9%) of the 33 isolates, respectively, that displayed a MEM > MEV phenotype. In the control group, these genes were overexpressed among 3 (30.0%), 7 (70.0%), 6 (60.0%), and 5 (50.0%) of the 10 MEM = MEV control isolates, respectively.

When the expressions of *mexX* and *mexXY* were combined as a single result due to the presence of these genes in the same operon, 30 (90.9%) isolates of the MEM > MEV group and 6 (60.0%) isolates of the MEM = MEV group exhibited overexpression of the MexXY efflux system. Moreover, when MexXY and PDC overexpression were analyzed together, 17 (51.5%) of the isolates in the MEM > MEV group had both genes overexpressed compared to 1 (10.0%) isolate from the MEM = MEV group.

**Fig 2:**
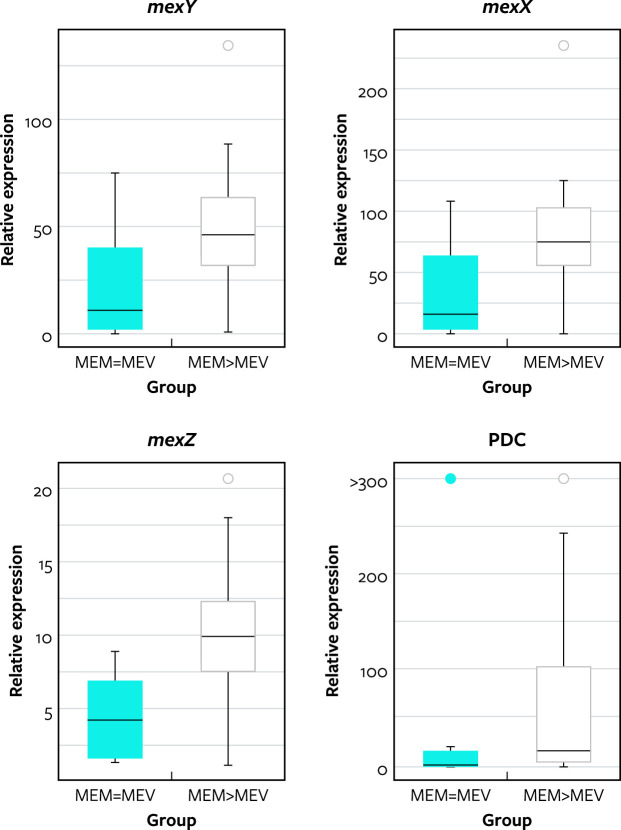
Relative expression of *mexY, mexX*, *mexZ*, and PDC from isolates displaying the same MIC values for meropenem and meropenem-vaborbactam (MEM = MEV) compared to meropenem-resistant and meropenem-vaborbactam-susceptible *P. aeruginosa* isolates (MEM > MEV).

Unfortunately, due to the small sample size (only 43 isolates), no statistical power was achieved to corroborate the differences noted in the presence/absence of features; however, the analysis of the expression rates for these genes demonstrated statistically significant higher levels of expression among the MEM > MEV group when compared to the MEM = MEV group (Fig. 2). The average expression of *mexX*, *mexY*, *mexZ*, and PDC was, respectively, 78.8/33.2X, 46.8/22.9X, 9.9/4.3X, and 222.4/119.0X, compared to the baseline for MEM > MEV/MEM = MEV isolates.

The other genes analyzed did not display differences among the two groups (Table 2), including *mexAB-oprM* overexpression detected among 16 (48.5%) and 5 (50.0%) isolates from the MEM > MEV and MEM = MEV groups, respectively. Disruption of OprD was noted among 29/33 (87.9%) of the MEM > MEV isolates and 9/10 (90.0%) of the MEM = MEV isolates (Table 2), and one additional isolate had reduced expression of OprD.

Analysis of mutations in genes encoding the known resistance contributors AmpR, ArmZ, MexZ, FtsI, DacB, DacC, NalD, and MexR did not reveal distinct amino acid substitutions among the MEM = MEV and MEM > MEV groups (Table 2).

## DISCUSSION

This study compared 33 *P*. *aeruginosa* isolates with confirmed meropenem MIC values greater than meropenem-vaborbactam MIC values (MEM > MEV) to 10 isolates with an MIC value at 8 mg/L for meropenem and meropenem-vaborbactam (MEM = MEV). The results revealed that a greater fraction of MEM > MEV than MEM = MEV isolates had an increased expression of the chromosomal *ampC* combined with an elevated expression of *mexXY*. Furthermore, the expression levels for the genes encoding AmpC (PDC), MexX, and MexY were significantly greater among the MEM > MEV phenotype isolates when compared to the MEM = MEV isolates. The generation of isogenic mutants with these characteristics was not conducted and is a limitation of the current study.

Riera et al. demonstrated that a combined *ampC* overexpression and *oprD* deletion increased meropenem MIC values four- to eightfold in a PAO1 background, conferring meropenem MIC values of 2 to 4 mg/L (20). These results are categorized as resistant when applying current breakpoint criteria that were developed using the standard dose of meropenem of 1 g q8h. Other studies evaluating clinical isolates demonstrated that when an *oprD* deletion is associated with *ampC* overexpression, a 32-fold increase was noted in the meropenem MIC values, leading to resistance (MIC 16 mg/L) even when applying a cutoff for 2 g q8h with an extended infusion that should cover MIC values up to 4 or 8 mg/L (21, 22). The approved standard dose of meropenem-vaborbactam is 2 g meropenem plus 2 g vaborbactam with a 3 h infusion, q8h.

In a study by Masuda et al., the overexpression of *mexXY* in isolates that already expressed high levels of *ampC* in a *mexAB-oprM* mutant background generated a fourfold increase in meropenem MIC values (5). It can be hypothesized that if the number of meropenem molecules inside the cell is constantly reduced by extrusion by this efflux system, then the AmpC (PDC) molecules could slowly hydrolyze meropenem, preventing its activity. In this case, vaborbactam would bind to the PDC molecules and protect meropenem from hydrolysis, which would explain the lower MIC values for meropenem-vaborbactam in the MEM > MEV group.

Lastly, this study indicated that when MIC values for meropenem-vaborbactam are lower than the results for meropenem alone, testing should be repeated for confirmation, as these results were not confirmed in 62.5% of the cases. While the disparity between MIC values for meropenem and meropenem-vaborbactam is not well appreciated, clinicians and microbiologists should be aware of such discrepancies. Meropenem-vaborbactam testing should be considered for *P. aeruginosa* isolates as populations of the genotypes observed here may be enriched in some settings.
